# Can a local low-budget intervention make a difference to suicide rates? Evaluating the effectiveness of the Barnet (London) suicide prevention campaign using real-time suspected suicide data

**DOI:** 10.1186/s12889-025-24553-8

**Published:** 2025-10-06

**Authors:** Bastien Chabé-Ferret, Lisa Marzano

**Affiliations:** Middlesex University, London, UK

**Keywords:** Suicide, Community-based interventions, Population-based interventions, Public health, Awareness campaigns, Peer support, Real-time suicide surveillance, Apps, Mhealth

## Abstract

**Background:**

Three quarters of suicides in the UK are by men, of whom only a quarter had contact with mental health services at the time of their death. Community-based interventions are therefore likely to be crucial to reduce (male) suicides, but there is limited evidence to support their effectiveness. The aim of this study was to evaluate the impact of a multi-strategy campaign to increase uptake of mental health services and peer support amongst working-aged men in Barnet, London, via: (1) targeted promotion of the ‘Stay Alive’ app, (2) a large scale digital and outdoor media campaign, (3) community outreach targeting male-dominated industries, (4) the first face-to-face “Andy’s Man Club” peer-to-peer support group in the borough.

**Methods:**

We used data on suspected suicides in London between 1st March 2021 to 31st November 2023 (*N* = 1,408) to calculate monthly age-standardised rates in (a) Barnet, (b) surrounding boroughs and (c) the rest of London, for ‘naïve’ and ‘placebo’ comparisons during and outside the campaign period, and then before, during and after the campaign. We also estimated maximum exposure to the campaign beyond its duration, and repeated the analysis using a more conservative (February to December 2020) baseline period for Barnet.

**Results:**

There was a sizeable drop in suicides in Barnet for the duration of the campaign and the following six months, with 6 to 9 deaths possibly averted thanks to the campaign, which represents a decline of around 20% of the yearly incidence, at a cost of under £6,400 per averted suicide.

**Conclusions:**

Our analysis suggests that a local, relatively inexpensive community-based campaign can be effective in reducing (suspected) suicides. However, further research is needed to confidently link this decrease in suicides to the campaign, or specific elements of it.

**Supplementary Information:**

The online version contains supplementary material available at 10.1186/s12889-025-24553-8.

## Introduction

Over 6,000 suicides are recorded annually in the UK [1]. Evidence that in only a quarter of cases the person had contact with mental health services at the time of their death [2] has spurred growing interest and investment in community-based measures to prevent suicide. For example, in England the government has recently made available a £10 million grant fund to support suicide prevention activities delivered by voluntary, community or social enterprise (VCSE) organisations [3], following the example of countries such as Australia and the US, where more established government-funded community-based programs have been shown to be effective in reducing suicide rates [4, 5].

Well-structured, coordinated community-based initiatives are held internationally as a crucial component of national suicide prevention strategies, as they can target key diagnostic and treatment gaps (particularly in relation to depression [6]) and enable an integrated approach at a local level, with protective benefits for families and individuals [7]. However, evidence of their effectiveness remains limited, inconsistent and overall weak [8–10]. For instance, in the UK there has been no formal evaluation of the Government’s first VCSE suicide prevention fund (2020-21) and internationally there is insufficient or unclear evidence to assess the benefits of community-based multi-strategy interventions [11] or of initiatives such as public awareness and education campaigns [12], gatekeeper training [13], crises lines [14], media guidelines [15], and peer support [16] interventions to reduce suicides. Robust evaluation of such measures has been hindered by the paucity of randomised controlled trials, and difficulties in assessing before and after impacts on suicidal behaviour (vs. attitudinal or other proxy or intermediate measures) because such data are often not available or sufficient for powered statistical analyses [12]. This is particularly an issue in relation to more localised interventions, where a low base rate of suicides makes it difficult to identify significant effects, and for reasons of poor capacity, for smaller and under-resourced interventions. 

In England, where official suicide data are based on coronial data, the recording of suicides by date of registration (rather than date of death), has further impeded evaluation of suicide prevention initiatives (the Office for National Statistics (ONS) estimates that around half the registered deaths reported in one year actually took place in the previous year [17]). However, the recent development of near to real-time surveillance systems (RTSS) to monitor suspected suicides (by date of death, and without the substantial time-lag of official suicide statistics) opens up new – but as of yet untapped - opportunities to examine the impact of community-based measures (and indeed other strategies) on suicide rates [18].

We used near-real time data on suspected suicides in London to examine borough-level variations in rates following a local campaign to prevent mental ill-health and suicidality amongst working-aged men. This multi-strategy intervention sought to encourage men (and others) to talk about their mental health, seek help and support other men in their lives. An internal, mixed-methods evaluation found that the campaign had been successful in terms of increasing awareness of local mental health resources and help-seeking, and had contributed to destigmatising mental health and suicide amongst men in the local community (the north London borough of Barnet, thereafter referred to as ‘Barnet’) [19]. The aim of this second, independent evaluation was to investigate whether, in addition to these important outcomes, the campaign was successful in reducing suicides in the borough.

## Methods

### The Barnet suicide prevention campaign

The Barnet suicide prevention campaign run between October and December 2021, and consisted of four interlinked elements:

1. Targeted promotion of the “Stay Alive” app [20], to signpost men (and others) to local and national support services, as well as useful information and tools to help stay safe in crisis.
2. A large scale digital and outdoor media campaign, with a focus on awareness building and urging local residents to download the Stay Alive app for themselves or to help others in their lives.
3. Community outreach targeting male-dominated industries, to raise awareness of mental health support and suicide prevention, and encouraging attendees to undertake the Zero Suicide Alliance training [21].
4. The first face-to-face “Andy’s Man Club” peer-to-peer support group in the Borough [22], to provide a space for men to speak openly about their mental health in a non-clinical environment.

In 2022 each component of the campaign was individually evaluated by the Barnet Public Health Directorate. The results showed that digital promotion of the Stay Alive app reached over 100,000 people in Barnet and that in the 3 months of targeted promotion (October to December 2021) there had been a 27% increase in new users in London and 5% increase in men in London using the *StayAlive *app, as well as increased engagement with local/Barnet resources on the app’s ‘Find Help Now’ section, and in website traffic to the app’s website. User satisfaction with the app was found to be positive, as was feedback from Andy’s Man Club attendees. Please see [19] for further details of the campaign’s development and initial evaluation.

### Design

We evaluated the impact of the campaign on suicide rates in Barnet as a natural experiment, comparing incidence rates adjusted for age, sex and seasonality, before, during and after the campaign. We additionally used data on the number of clicks to services to estimate a plausible longer-term of effect. We analysed these estimates in regards of two comparison groups: (1) immediately adjacent boroughs (Brent, Camden, Enfield, Haringey and Harrow - to check for potential ‘spillover effects’) and (2) all other London boroughs (all based on borough of residence). Table 1 presents a set of descriptive statistics showing that Barnet had a slightly smaller share of working age population than other areas in London, a similar sex ratio, a slightly larger incidence of male suicides, and an incidence of female suicide that lied between that of boroughs immediately around and that in the rest of London. We finally ran two placebo exercises to test the robustness of our findings. Natural experimental methods are a well-established alternative to randomised designs to evaluate the effects of specific changes, policies and interventions on suicide rates [23].

**Table 1:** Population-level descriptive statistics for Barnet and comparison areas

	Barnet	Around Barnet	Rest of London
Population	324,012	1,187,266	5,907,281
Share of 18–65 population	75.0%	77.6%	79.4%
*Sex ratio (male to female)*	*0.91*	*0.92*	*0.93*
*Male annual suicide incidence (per 100*,*000)*	*10.16*	*9.10*	*9.93*
*Female annual suicide incidence (per 100*,*000)*	*2.87*	*2.29*	*3.72*

### Suspected suicides data and rates

The analysis is based on suspected suicides recorded between March 1 st 2021 and November 30th 2023 in the pan-London real-time surveillance system, the Thrive LDN RTSS (*N* = 1,408). Anonymised information about the deceased age, sex, borough of residence and date of death was made available to the research team, and used to select all cases where the individual had resided in London at the time of their death (*N* = 1,336, 94.9% of all suspected suicides recorded in London over the study period).

We then merged this information with data on population by age, sex and borough of residence from the 2021 Census [24] to derive rates of suspected suicide (per 100,000 population) for the three ‘treatment groups’: (1) Barnet, (2) Barnet surroundings (Brent, Camden, Enfield, Haringey and Harrow) and (3) the rest of London. In particular, we calculated age-specific monthly incidence rates by sex and borough of residence $\:{\lambda\:}_{asbm}$ by dividing the number of suicides for a given month $\:m$, age group $\:a$, sex $\:s$ and borough of residence $\:b$ by the total population in that category in the 2021 Census. We then age-standardised those rates by taking a weighted average of all rates for a given sex and borough of residence, where weights are given by the ratio of the total London population in each age group and sex $\:{Pop}_{as}$ over the total London population of that sex $\:{Pop}_{s}$ according to the following formula:

$$\:{{\Lambda\:}}_{sbm}={\sum\:}_{a}\frac{{Pop}_{as}}{{Pop}_{s}}{\lambda\:}_{asbm}$$

In a second phase of the analysis, we extended the RTSS data for Barnet back to February 2020. As this information was not broken down by sex and age, we assigned all suspected suicides in that period (*N* = 13) to males aged 45–54, the highest risk group.

### Analysis

Data were first inspected visually, using Kaplan-Meier failure curves. Moving on to the monthly incidence rates, we checked the stationarity of our series of interest using an augmented Dickey–Fuller test for unit root. We rejected non-stationarity for monthly suicide rates in Barnet for both men and women at the 5% level. We then performed two “naïve” comparisons: incidence rates during and outside the campaign period, and then before, during and after the campaign. To that end, we estimated the following econometric models:

1 $$\begin{aligned}\:{\Lambda\:}_{sbm}=&\,{\beta\:}_0+\:{\beta\:}_1treatment_b+{\beta\:}_2campaign_m\\&+{\beta\:}_3campaign_m\times\:treatment_b\\&+{\Gamma\:}_s+{\beta\:}_4\text{c}\text{o}\text{s}(2\times\:\pi\:\times\:month_m/12)\\&+{\beta\:}_5\text{s}\text{i}\text{n}(2\times\:\pi\:\times\:month_m/12)\\&+\varepsilon_{sbm} \end{aligned}$$

2 $$\begin{aligned}\:{\Lambda\:}_{sbm}=&\:{\beta\:}_0+\:{\beta\:}_1treatment_b+{\beta\:}_2campaign_m\\&+{\beta\:}_3campaign_m\times\:treatment_b+{\beta\:}_2^{'\:}post_m\\&+{\beta\:}_3^{'\:}post_m\times\:treatment_b+{\Gamma\:}_s\\&+{\beta\:}_4\text{c}\text{o}\text{s}(2\times\:\pi\:\times\:month_m/12)\\&+{\beta\:}_5\text{s}\text{i}\text{n}(2\times\:\pi\:\times\:month_m/12)+\varepsilon_{sbm}\end{aligned}$$

Where $\:{{\Gamma\:}}_{s}$ is a fixed effect for sex, seasonality is controlled for by the inclusion of trigonometric terms capturing the annual cyclicality, and $\:\varepsilon_{sbm}$ is the error term [25, 26]. We tested the presence of seasonality in the data by running an F-test on the joint significance of $\:{\beta\:}_{4}$ and $\:{\beta\:}_{5}$. With a value of 9.94, we strongly reject that the coefficients capturing seasonality are jointly equal to zero. Controlling for seasonality is crucial in our context as the period of the campaign corresponds to the usual trough in the suicide cycle. Failing to do so may therefore lead to overestimating the effect of the campaign.

The variable $\:campaign$ takes value 1 for the months of October, November, and December 2021, and 0 otherwise. The variable $\:post$ takes value 1 for all months after December 2021, and 0 otherwise. $\:treatment$ is a categorical variable taking value 1 for Barnet residents, 2 for around Barnet and 0 for the rest of London. We estimated these models by Ordinary Least Squares (OLS) and calculated the mean incidence they predict for all possible values of treatment and campaign, which allowed to neutralise the potential effects of sex and seasonality.

Even though the campaign was targeted at middle-aged men, we chose to use the overall age-adjusted incidence of suicide of men and women in a given borough as our main dependent variable. This is because suicide at the borough-month level is a rare event, resulting in very volatile series. Examining incidence for both men and women allows to smoothen those series, therefore improving the precision of our estimations. On the other hand, this approach presents the risk of diluting the effect of the campaign by including a population that was not explicitly targeted. For transparency, we replicated our analysis on the sample of men only and provide them in Appendix A. We find that the results are qualitatively similar, though slightly larger in magnitude, at the cost of a lower level of significance. If anything, the results we provide in the cost-benefit analysis are therefore conservative.

Using ‘click’ data (i.e. the number of clicks on local and locally-available support resources via the campaign’s app) we also estimated exposure to the campaign beyond the duration of the campaign itself (October to December 2021), to explore the possibility of effects lasting longer than this three-month period. We therefore created a measure of exposure to the campaign that contained the fraction of time one has been exposed to the campaign over the past six months. As shown in Fig. 2, our exposure measure starts increasing on the first month of the campaign, reaches its peak after 3 months of exposure over the last 6 (so 50% exposure), and start diminishing when 6 months have passed since first exposure. The idea was to capture the cumulative effect of exposure to the campaign over time, as well as the slow fading away of effects after its end. The exposure variable was therefore used to estimate the following equation, to predict monthly incidence rates across treatment groups:

3 $$\begin{aligned}\:{\Lambda\:}_{sbm}=&\:{\beta\:}_0+\:{\beta\:}_1treatment_b+{\beta\:}_2exposure_m\\&+{\beta\:}_3exposure_m\times\:treatment_b\\&+{\Gamma\:}_s+{\beta\:}_4{\beta\:}_4\text{c}\text{o}\text{s}(2\times\:\pi\:\times\:month_m/12)\\&+{\beta\:}_5\text{s}\text{i}\text{n}(2\times\:\pi\:\times\:month_m/12)\\&+\varepsilon_{sbm}\end{aligned}$$

For each comparison, we estimated (1) the likely number of averted suicides; (2) whether the campaign had a statistically significant impact on suicide rates in Barnet; (3) cost-effectiveness, measured as the cost of each averted suicide, based on the price of the entire campaign (£39,355, excluding in-kind contribution of Council officers’ time).

As suicides in Barnet appeared to be high in the immediate pre-campaign period, we also repeated the analysis using a February to December 2020 baseline (i.e. excluding January to September 2021)^1^. This was intended to produce a more conservative estimate of the campaign’s impact on suspected suicides in Barnet. Indeed, this is likely to have been a particularly conservative baseline, as age-standardised rates of suicide for England were lower at the start of the Covid-19 pandemic and associated restrictions (April to December 2020) than the same period in 2019 and 2018 [27].

### Robustness checks

We carried out two ‘placebo exercises’ to estimate the amount of caution required in interpreting the results of our analyses. The first one consisted in estimating the impact of exposure to the campaign on each individual borough as if they had all been equally exposed. This was to assess how likely we were to pick up a false positive by estimating the effect of our exposure variable on areas that have not been treated. The second one consisted in estimating the impact of three placebo campaigns of the same length that would have hypothetically run in each borough a random number of months after the one that was actually run in Barnet. This enabled us to verify how frequently we would obtain significant estimates using the shape of our exposure measure over boroughs and periods for which we know they would be false positives.

## Results

**Fig. 1:**
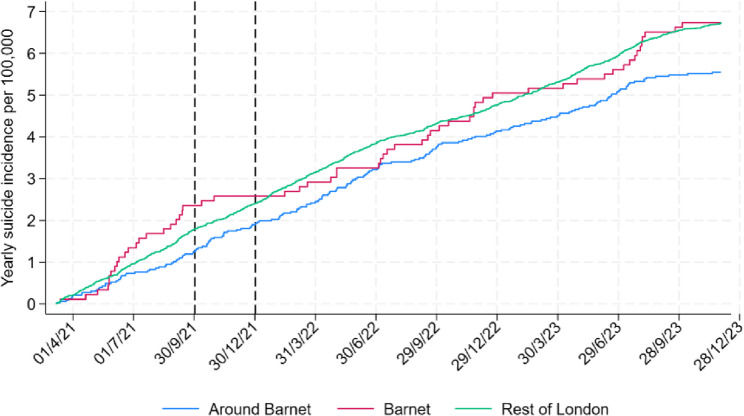
Timing of suicides across treatment groups. Note: This figure shows the cumulative number of suicides per 100,000 population over the study period. The curves reach the total yearly suicide incidence by the end of period. The slope of each curve at any point represents the instantaneous suicide incidence rate at that time

Figure 1 below shows the pace at which suicides occurred in the three treatment groups to reach the overall yearly incidence over the study period (1st March 2021 to 30th November 2023).

**Fig. 2:**
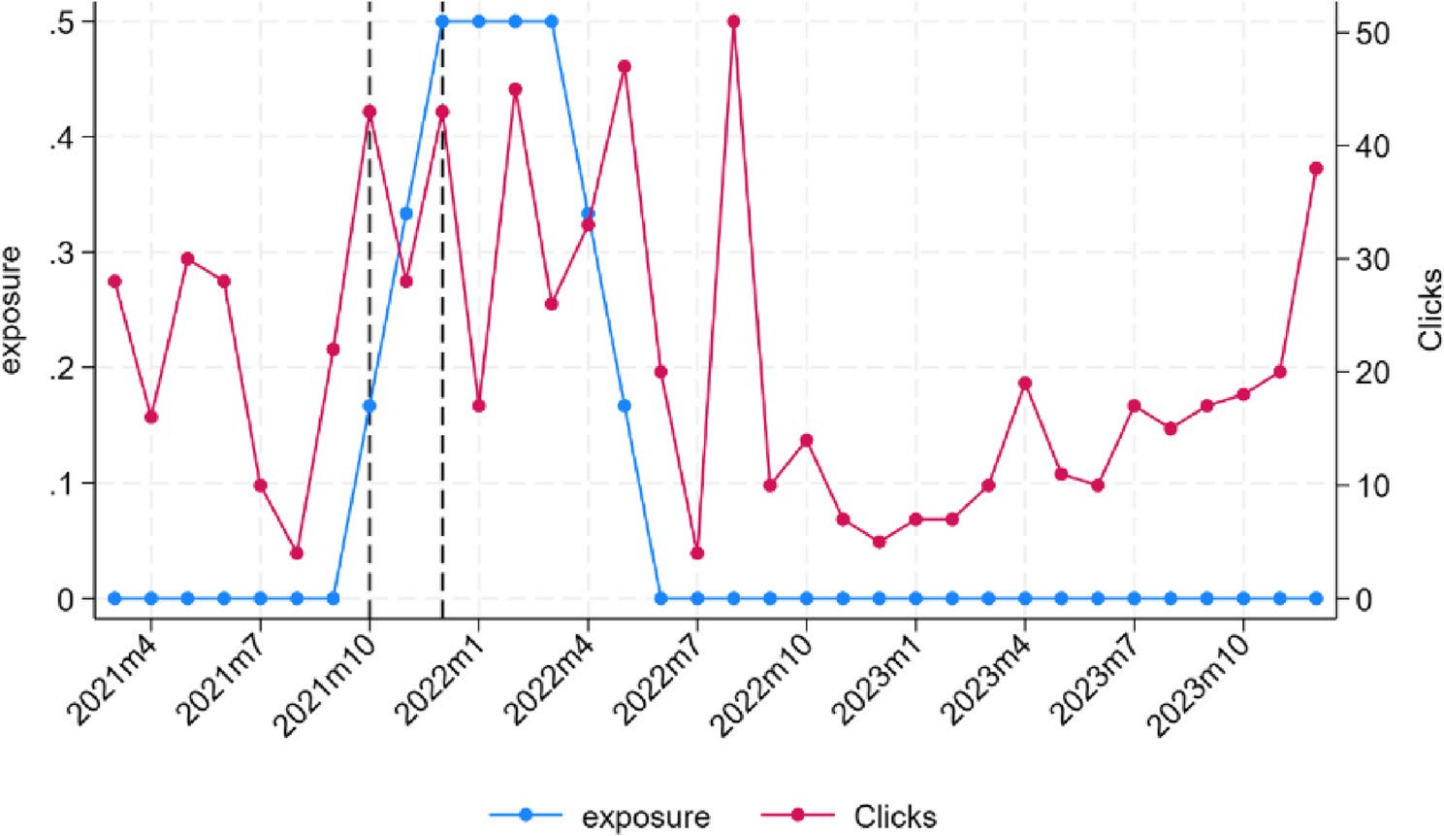
Evolution of clicks to services and measure of exposure. Note: Exposure is calculated as the fraction of time an area has been exposed to the treatment in the previous 6 months. The maximum value of 0.5 reflects periods a total exposure of 3 months in the last 6 (50%)

Visual inspection of these data suggests that:

1. The overall yearly suicide rate was just below 7 suicides per 100,000 population, both in Barnet and the rest of London. It was substantially lower in the Barnet surroundings at around 5.5.
2. In the four months leading up to the campaign, this pace was higher in Barnet than in the rest of London, while it was slightly lower in the Barnet surroundings.
3. During the course of the campaign (October to December 2021), the pace at which suicides occurred in Barnet slowed down markedly (there were no suspected suicides amongst Barnet residents in November and December 2021, or in January 2022), while it increased somewhat around Barnet. The pace does not seem to have changed significantly for the rest of London.
4. The pace remained lower in Barnet for about six months after the intervention. This period also coincided with a period of continued engagement with the campaign’s suicide prevention app. Analysis of ‘click data’ showed a substantial increase in clicks to local support service from October 2021 (when the campaign was launched) to June 2022 (6 months after it ended) (shown in blue in Fig. 2 below).

**Fig. 3:**
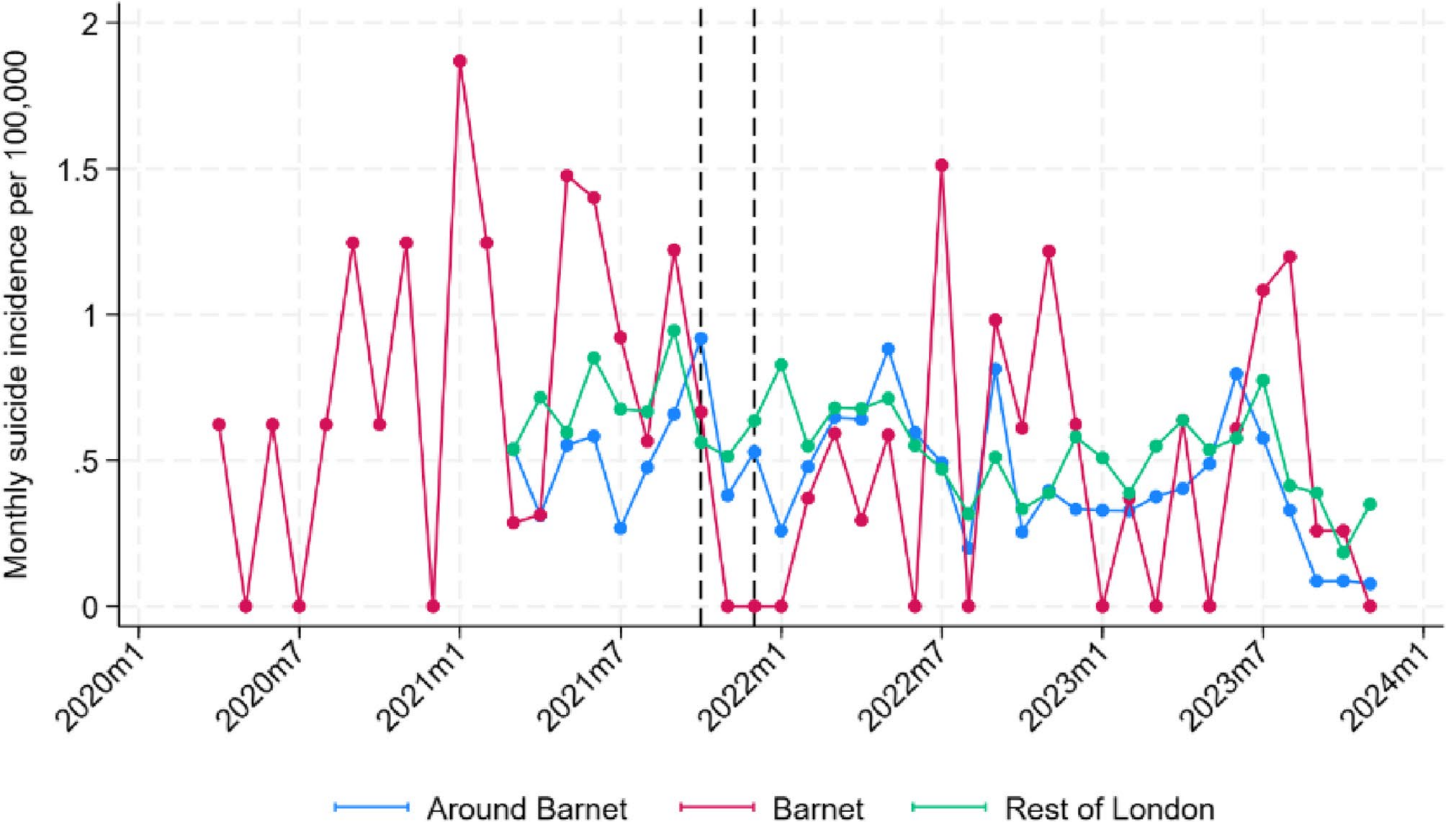
Age-standardised incidence rates across treatment groups

**Fig. 4:**
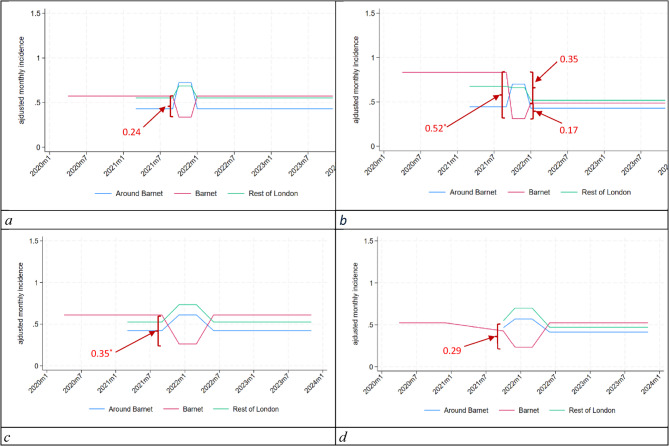
**a** Comparisons of monthly incidence across treatments during versus off-campaign. **b** Comparisons of monthly incidence across treatments pre-, during and post-campaign. **c** Estimation of lasting effects of the campaign. **d** Lasting effect with conservative baseline

Figure 3 depicts age-standardised monthly incidence rate by treatment groups, whilst Fig. 4 displays differences:

- ‘during and outside’ the campaign period (i.e. October to December 2021 vs. all remaining months in the study period);
- before, during and after the campaign (i.e. March to September 2021; October to December 2021; January 2022 to November 2023);
- based on the campaign’s estimated lasting effects (i.e. October 2021 to June 2022);
- similar to c) but using a February to December 2020 baseline (as a more conservative ‘control’ period/baseline than March to September 2021).

Comparison of campaign on vs. off periods (Fig. 4a) suggest a 10% drop of the yearly incidence of suspected suicides, implying that about 3.52 suicides may have been averted.^2^ This decrease, however, was not statistically significant (during the campaign, the incidence of suspected suicides dropped by 0.24 (CI: [−0.68;0.21]; *p* = 0.30) per 100,000 population). Over the same period rates of suspected suicides rose by 0.29 (CI: [−0.005;0.59]; *p* = 0.054) per 100,000 population in boroughs around Barnet. This rise is just short of being statistically significant at the 5% level. There was also a slight increase in incident rates in the rest of London of 0.13 (CI: [0.004;0.26]; *p* = 0.043) per 100,000 population, which was statistically significant at the 5% level. Both observations make a case against any positive spillovers of the campaign to surrounding areas.

The drop in suicides rates in Barnet during the campaign period was larger in magnitude and statistically significant at the 10% level when considering differences across three periods: pre, during and post-campaign (−0.52 (CI: [−1.10;0.060]; *p* = 0.079); see Fig. 4b). This 3-month drop implies a 23.1% decrease in the yearly incidence, or about 7.6 averted suicides^3^. The figure suggests a long-term impact of −0.35, which fails to be statistically significant at conventional levels.

Maximum exposure to the campaign was associated with a decrease in incidence of 0.35 (CI: [−0.70;0.01]; *p* = 0.057) suicides in Barnet per 100,000 population (Fig. 4c). This decrease was just short of statistical significance at the conventional 5% level and can explain a drop in yearly incidence by 26% of the baseline, or about 8.55 suspected suicides^4^. In other words, the overall impact of the campaign is about two and half times larger when considering its lasting effects (based on continued engagement with the campaign app for six months beyond the formal end of the campaign (Fig. 2)) than when taking into account the campaign period alone.

However, when using a more conservative baseline period (Fig. 4d) we obtained a drop in incidence of 0.29 (CI: [−0.67;0.09], *p* = 0.129) at maximum exposure. This is smaller than the effect estimated using the year 2021 in the baseline, but remains large in magnitude and just short of statistical significance at the 10% level. Overall, it represents a decrease in yearly incidence of 19%, or 6.21 suicides averted.^5^

The results of these analyses are summarised in Table 2, alongside crude cost-effectiveness estimates obtained by dividing the total cost of the campaign by the estimated number of averted suicides.

**Table 2:** Cost-effectiveness estimates of the Barnet suicide prevention campaign

Method	Suicides averted	Statistical significance	Cost-effectiveness^1^
a) Campaign on/off	3.52	No	£11,180
b) Campaign pre/on/post	7.62	Yes (w/reservations)*	£5,165
c) Campaign w/lasting effects	8.55	Yes (w/reservations)*	£4,602
d) Campaign w/lasting effects & conservative baseline	6.21	No	£6,337

*p < 0.10

^1^The entire campaign was costed at £39,355, excluding Council officers’ time. We divided this sum by the number of suicides averted by the campaign according to our calculations

### Placebo exercises

**Fig. 5:**
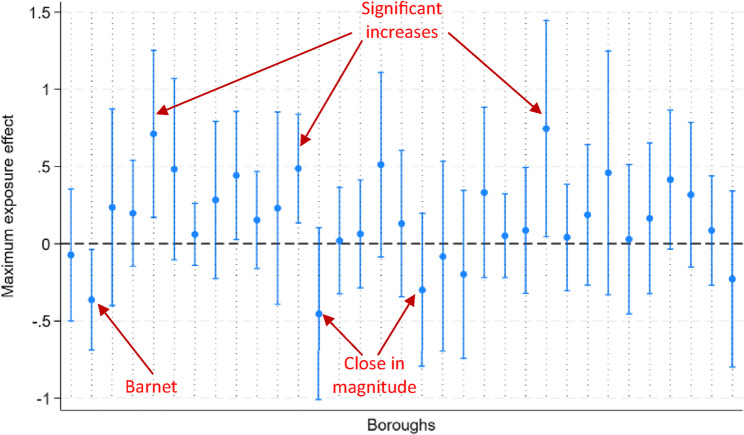
Effect of Barnet campaign on all boroughs

We estimated the impact of exposure to the campaign on each individual borough as if they had all been equally exposed (Fig. 5). Barnet was the only borough for which we found a statistically significant decrease in the incidence of suicides associated with exposure to the campaign. However, we identified impacts that are similar in magnitude for two other boroughs, though not statistically significant at conventional levels. Furthermore, three other boroughs (out of 32) saw their incidence increase significantly above their long-run value.

**Fig. 6:**
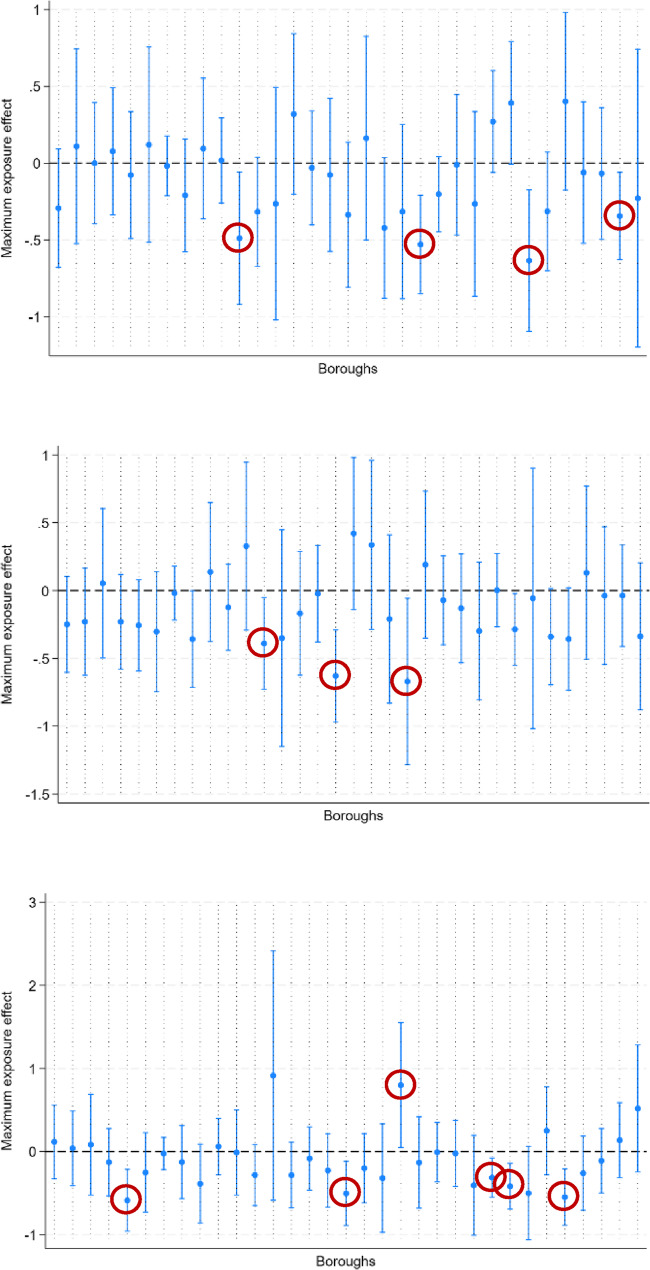
Estimation of placebo interventions at different times

We also estimated the impact of a placebo campaign that had hypothetically run after the one that was actually run in Barnet (Fig. 6). In 12 instances (circled in red), the placebo treatment had a significant impact on lowering suicide rates (out of 96 estimations in total). This suggests a rate of false positives of 12.5%.

## Discussion

Using near to real-time data on suspected suicides, we investigated monthly variations in age-standardised suicide rates in relation to a multi-pronged community-based campaign to reduce suicides in Barnet (vs. rates in neighbouring boroughs and the rest of London). Our analysis suggests that the campaign may have had an impact on suicide rates in Barnet for the duration of the campaign itself and the subsequent six months – potentially helping save over eight lives during this nine-month period, at minimal cost.

As this estimate may (also) capture a return to Barnet’s ‘normal’ baseline after a high incidence period immediately prior to the campaign, we also calculated a more conservative estimate (based on an earlier, more conservative baseline period). The results of this conservative analysis suggest that six lives were possibly averted thanks to the campaign in the nine-months from its launch in October 2021, at a cost of about £6,000 per averted death. This effect remains large in magnitude but failed to reach significance at the 10% level.

Based on this estimate, our results suggests that the intervention in Barnet was as cost-effective as interventions more directly targeted to at-risk populations [28, 29]. However, a more comprehensive cost-benefit analysis is needed to confirm this.

### Limitations

Our crude cost-effectiveness estimate did not include in-kind contributions or staff time and is therefore an imperfect proxy for the real cost of the campaign. Furthermore, the campaign was not part of a randomised control experiment, and the possibility of contextual differences limits the generalisability of our findings. For example, it is worth noting that our analysis spans over different phases of the Covid-19 pandemic and associated restrictions, and subsequent cost of living crisis. Whilst these affected all the areas being compared, we cannot exclude that the campaign may have had a different impact under different circumstances.

The results of our ‘placebo exercises’ also suggest a moderate level of confidence in strictly causal interpretations of these results, given the relatively high rate of false positive (12.5%) obtained when estimating the simulated effect of campaigns running in subsequent periods after the actual intervention.

### Implications

This study adds to the small but growing literature on the effectiveness of community-based strategies to prevent suicide, including awareness campaigns [30], suicide prevention apps [31], and peer support for suicide prevention [16]. Our findings suggest that there was a sizeable drop in suicides in Barnet during and after the campaign, which is consistent with (some) previous studies [10] and with the positive evaluation results reported in Barnet in 2022 (e.g. in terms of increased awareness of local mental health resources and help-seeking behaviours, and in relation to the campaign’s reach and positive user feedback [19]). However, we cannot firmly exclude that this decrease in suicide rates happened by chance or confidently link it to the campaign (or to specific elements of the campaign), because of the aforementioned limitations. One way of addressing these questions could be to repeat the campaign (or elements thereof) at a different time point and/or in different borough/s or local authority/ies, ideally selected on a random or quasi-random basis.

Our study also demonstrates the feasibility of using near-real time data to examine borough-level variations in (suspected) suicides following a specific intervention or other change. However, it is important to point out that our analyses were based on *suspected* suicides, so any inferences or conclusions about *suicides* should be made cautiously, and the possibility of missing or inaccurate data cannot be excluded. Albeit time and resource intensive, review and validation of such data after coronial inquest could help address important questions about data quality, completeness and sensitivity. Recent research suggests that suspected suicide data from established RTSSs can accurately reflect suicide rates confirmed by official statistics [32].

Analysis of RTSS data could be complemented and triangulated using attempted suicide data (now also being monitored near-real time in London, and other parts of the country) and other indicators of help-seeking (e.g. calls/self-referrals to support services and helplines). National (as well as London-wide) comparisons could be explored, using near to real-time suspected suicide surveillance (nRTSSS) data for England [33] and police-led nRTSSS data for Great Britain [18].

Qualitative research with people with lived/living experiences of suicide, and those around them (including family and friends, and service providers), can provide crucial insights into *what* can be effective in preventing suicide, as well as *how*,* why*,* when* and *for whom*. Further follow-up research could seek to explore from these important perspectives the longer-term reach and impact of different elements of this campaign, including the possibility of unintended consequences or oversights, and how to maximise the impact of any future campaign and the likelihood of long-lasting effects. Such an analysis could extend to examination of relevant discussions and interactions in a range of online spaces, including ‘pro-choice’ suicide sites and social media [34].

## Conclusions

This is the first study to utilise near to real-time data on suspected suicides in London to examine the impact of a multi-component suicide prevention campaign targeting working-age men in Barnet. Aimed at increasing uptake of local mental health services and peer support, the Barnet campaign appears to have had lasting effects beyond the duration of the campaign itself (October to December 2021). Our analysis shows that, compared to neighbouring boroughs and the rest of London, there was a sizeable drop in suspected suicides in Barnet for the duration of this intervention and the following six months. Our preferred, most conservative estimate indicates that six suicides were possibly averted thanks to the campaign, at a cost of about £6,000 per averted death.

However, we cannot firmly exclude that this decrease in suicide rates happened by chance or confidently link it to the campaign, or to specific elements of the campaign. Further research is needed to explore these important questions, ideally using random or quasi-random designs, comprehensive cost-benefit analyses, and a focus on the perspectives of those who may benefit (or not) from interventions such as these.

## Supplementary Information

Supplementary Material 1.

## Data Availability

Currently data access is restricted, although aggregate anonymised data collected could be made available with the permission of the Barnet Public Health Directorate and Thrive LDN.

## Footnotes

1 RTSS data indicate a 0.98 monthly incidence on average for January to September 2021, corresponding to an 11.76 (per 100,000 population) yearly incidence vs. a rate of 0.55 from February to December 2020 (the more conservative baseline we suggest), or 6.64 expressed yearly. This is more in line with the yearly incidence of 7 observed overall for Barnet for 2021–2023 using RTSS data. Whilst there is no directly comparable official data based on ‘confirmed’ suicides, the conservative baseline we used is also closer to the yearly rate of 6 (per 100,000 population) reported by the ONS for 2018–2020 (based on date of registration) [35]).​

2 0.24*3(months)/6.74(average yearly incidence) = 10.7% and 0.24*3(months)*488,654(population in Barnet)/100,000 = 3.52.

3 0.52*3(months)/6.74(average yearly incidence) = 23.1% and 0.52*3(months)*488,654(population in Barnet)/100,000 = 7.62.

4 (0.35*3(months) + 0.23*2(months) + 0.12*2(months))/6,74(yearly incidence) = 26%, (0.35*3(months) + 0.23*2(months) + 0.12*2(months))*488,654(Barnet pop)/100,000 = 8.55.

5 (0.29*3(months) + 0.19*2(months) + 0.1*2(months))/6,74(yearly incidence) = 19%, (0.29*3(months) + 0.19*2(months) + 0.1*2(months))*488,654(Barnet pop)/100,000 = 6.21.
