# Supplementary material for: Can a local low-budget intervention make a difference to suicide rates? Evaluating the effectiveness of the Barnet (London) suicide prevention campaign using real-time suspected suicide data
Source: BMC Public Health. 2025 Oct 6;25:3350. doi: 10.1186/s12889-025-24553-8 (PMC12502295; doi:10.1186/s12889-025-24553-8)
Supplement: Supplementary file 1 — Supplementary Material 1. [file 12889_2025_24553_MOESM1_ESM.docx]

**Appendix A. Baseline results for men only**

| 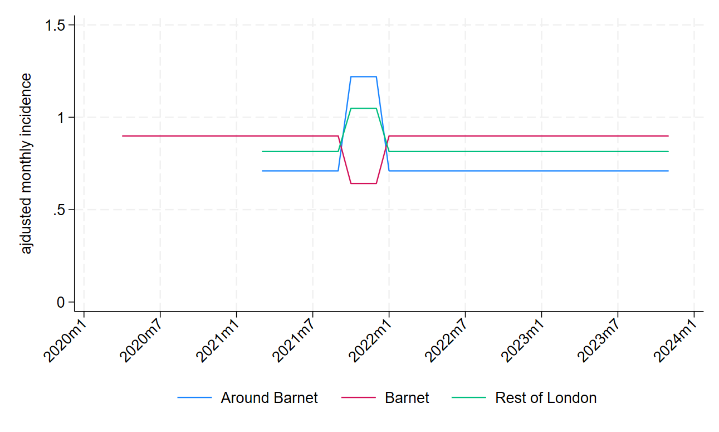  0.26 | 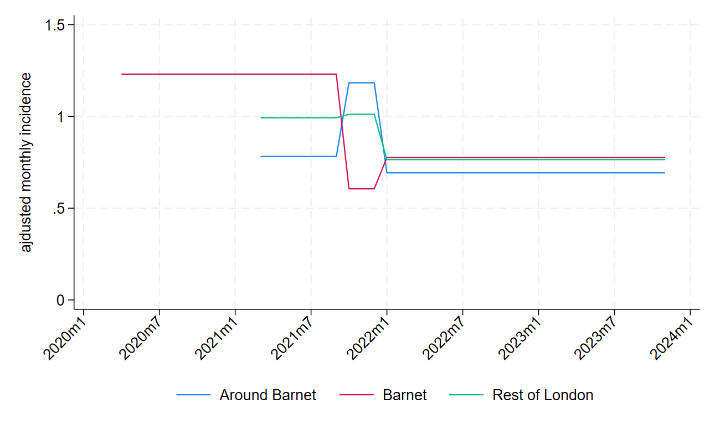  0.17  0.45  0.63 |
| --- | --- |
| *Figure A1a: Comparisons of monthly incidence across treatments during versus off-campaign (men only)* | *Figure A1b: Comparisons of monthly incidence across treatments pre-, during and post-campaign (men only)* |
| 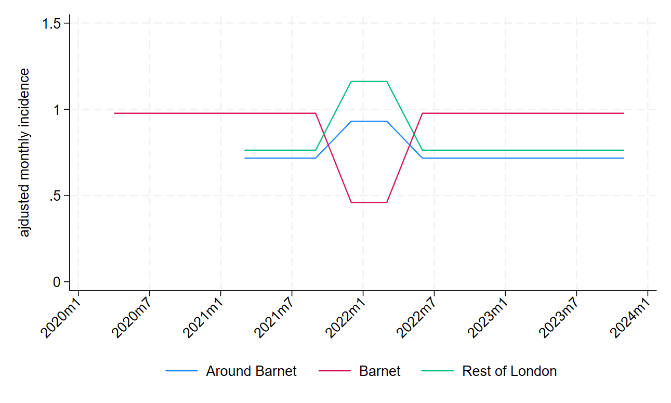  0.52 | 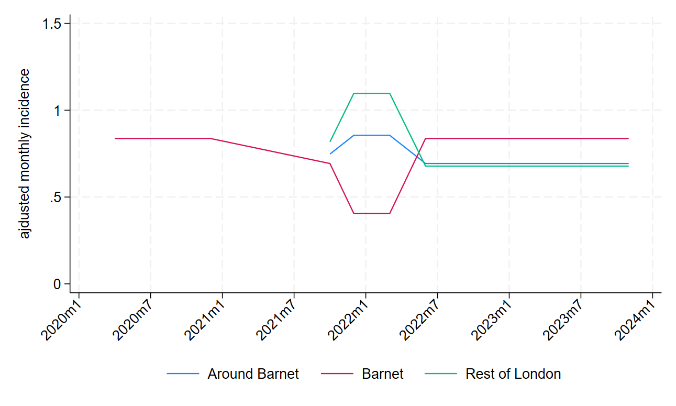  0.43 |
| *Figure A1c: Estimation of lasting effects of the campaign (men only)* | *Figure A1d: Lasting effect with conservative baseline (men only)* |
